# Frequency-dependent functional alterations in people living with HIV with early stage of HIV-associated neurocognitive disorder

**DOI:** 10.3389/fnins.2022.985213

**Published:** 2023-01-09

**Authors:** Wei Wang, Dan Liu, Yuanyuan Wang, Ruili Li, Jiaojiao Liu, Mingming Liu, Huasong Wang, Hongjun Li

**Affiliations:** ^1^Department of Radiology, Beijing Youan Hospital, Capital Medical University, Beijing, China; ^2^Department of Radiology, Renji Hospital, Shanghai Jiao Tong University School of Medicine, Shanghai, China; ^3^Department of Radiology, Beijing Second Hospital, Beijing, China; ^4^Department of Radiology, Xuanwu Hospital, Capital Medical University, Beijing, China; ^5^Physical Examination Center, Cangzhou Central Hospital, Cangzhou, Hebei, China; ^6^Department of Neurosurgery, Zhuhai People’s Hospital, Zhuhai, Guangdong, China

**Keywords:** HIV-associated neurocognitive disorder, functional MRI, voxel-mirrored homotopic connectivity, people living with HIV, amplitude of low-frequency fluctuation, regional homogeneity

## Abstract

**Background:**

HIV enters the brain soon after seroconversion and causes HIV-associated neurocognitive disorder (HAND). However, the pathogenesis of this insidious impairment at an early stage remains unclear.

**Objectives:**

To explore functional integration and segregation changes at the early stages of HAND, voxel-level indices of regional homogeneity (ReHo), the amplitude of low-frequency fluctuations (ALFF), and voxel-mirrored homotopic connectivity (VMHC) under two different frequency bands (slow-5: 0.01–0.027 Hz; slow-4: 0.027–0.073 Hz) were analyzed.

**Methods:**

Ninety-eight people living with HIV (PLWH) and 44 seronegative controls underwent resting-state functional magnetic resonance imaging. Furthermore, all PLWHs underwent neuropsychological and daily functioning tests. The main effect of the group and the interaction between the group and frequency band were investigated. Finally, the relationship between the altered indices and the cognitive domains was explored.

**Results:**

A significant group-by-frequency interaction was demonstrated in the right thalamus for ReHo; for VMHC, the interaction was observed in the bilateral precuneus and paracentral gyrus. The *post hoc* Bonferroni test indicated that the alteration of ReHo and VMHC could only be detected in slow-5. PLWH showed significantly reduced ALFF in both the frequency bands in the right occipital gyrus and right calcarine. Moreover, some altered functional integration and segregation indices are related to impaired cognitive function.

**Conclusion:**

People living with HIV displayed aberrant functional integration and segregation at the early stages of HAND, which is linked to cognitive function. The frequency band of slow-5 might be more sensitive for detecting insidious damage at an early stage.

## 1. Introduction

With advances in combination antiretroviral therapy (cART), the life expectancy of people living with HIV (PLWH) has approached that of the general population (van Sighem et al., 2010). Despite sustained viral suppression, the latent reservoir of the virus cannot be eradicated (Rojas-Celis et al., 2019; Wallet et al., 2019), and the risk of HIV-associated neurocognitive disorder (HAND) increased accordingly with increased cognitive impairment in multiple domains (Eggers et al., 2017; Winston and Spudich, 2020).

According to the Frascati criteria, HAND is divided into three categories: asymptomatic neurocognitive impairment (ANI), symptomatic mild neurocognitive impairment (MND), and HIV-associated dementia (HAD). For the diagnosis of MND and HAD, self-reported or knowledgeable others observed that everyday functioning decline is required, while in ANI, everyday functioning does not interfere (Antinori et al., 2007). In the cART era, the most severe form of HAND, that is HAD, has reduced dramatically, but milder forms are prevalent, especially ANI (Wang et al., 2020). As PLWH age, their cognitive impairment exacerbates and interferes with their daily life, including drug compliance (Woods et al., 2017; Gouse et al., 2021). Therefore, early detection and interference are important. While the commonly used Frascati criteria provide a uniform approach to the diagnosis of HAND, it requires neuropsychological tests in at least five cognitive domains from a qualified third party. The time-consuming nature and complexity of the assessment make these criteria difficult to implement widely in clinical practice. Moreover, differences in cognitive reserve may render neuropsychological tests insufficiently sensitive to detect early cognitive impairment.

In recent years, there has been increased interest in functional imaging in neuroHIV, especially resting-state functional magnetic resonance imaging (rs-fMRI), which maps the spontaneous neural activity at the macroscopic scale. Diverse analysis and processing methods allow us to study functional integration and segregation from different perspectives (Lv et al., 2018). The amplitude of low-frequency fluctuation (ALFF), regional homogeneity (ReHo), and voxel-mirrored homotopic connectivity (VMHC) are whole-brain, voxel-wise analytic approaches that can be used to explore the intensity of spontaneous activity of each voxel, the synchronicity of a particular voxel compared to its surrounding voxels, and the synchronicity of two mirrored voxels, respectively (Zang et al., 2004; Yang et al., 2007; Zuo et al., 2010b). Studies have shown that PLWH display abnormal ALFF and ReHo in the frontal lobe, the occipital lobe, the primary sensorimotor area, and the temporal lobe (Bak et al., 2018; Yadav et al., 2018; Li et al., 2019). These studies were not focused on PLWH at the early stages of HAND. Moreover, all studies were conducted within a traditional frequency band, while ignoring sub-frequency bands within this typically adopted band may have different properties and physiological implications (Zuo et al., 2010a; Chang et al., 2019; Yang et al., 2021; Zhang et al., 2021). Slow-4 and slow-5 oscillations were mainly detected within gray matter and were related to functional connectivity, while slow-3 and slow-2 oscillations were mainly restricted within white matter and were related to respiratory and cardiac signals (De Luca et al., 2006; Zuo et al., 2010a). Zuo et al. (2010a) showed that slow-4 was more robust in the basal ganglia, the thalamus, and the precuneus, while slow-5 was more robust in the medial prefrontal cortex. Subsequent frequency-specific studies demonstrated that functional alterations in Parkinson’s disease were more prominent in slow-4 (Liao et al., 2021), while in Alzheimer’s disease, slow-5 can be more sensitive (Han et al., 2011). However, more studies suggested the two frequency bands offer complementary information (Wu et al., 2020; Yang et al., 2021).

We used slow-5 and slow-4 in our study to explore functional segregation (ALFF) and integration (ReHo and VMHC) in different frequency bands in PLWH with early cognitive impairment. The combined use of these three indices in different frequency bands may potentially reveal the specific brain regions affected in PLWH at the early stage of HAND. We also investigated the relationship between altered indices and neurocognitive functions, which may provide new insights into the pathogenesis of HAND.

## 2. Materials and methods

### 2.1. Subjects

The study was conducted in accordance with the Declaration of Helsinki, and the protocol was approved by the Ethics Committee of Beijing You’an Hospital. Signed informed consent was obtained from all patients before any study-specific procedure was performed. Ninety-eight HIV-seropositive individuals from the You’an Hospital’s STD/AIDS Clinic and 44 seronegative controls from communities were recruited. For seronegative controls, we recruited age-matched participants using flyers. The inclusion criteria are as follows: 1. Confirmed HIV infection by PLWH treatment qualified institutions; 2. For PLWH, no self-reported or knowledgeable informant-reported acquired everyday functioning impairment for primary screening to exclude those with MND or HAD; 3. Volunteered to participate in the study and signed a written informed consent; 4. Right-handed. The exclusion criteria are as follows: anxiety and depression disorder, obsessive–compulsive disorder, history of head trauma or coma, or history of drug or alcohol abuse.

### 2.2. Neuropsychological assessment

In this study, detailed neuropsychological examinations were conducted in six cognitive domains using the validated Chinese version of the psychological tests normed for age, years of education, and residence scale (Shi et al., 2015). The neuropsychological suite contains nine subtests, including, 1. Speed of information processing [trail-making test part A (TMT A)]; 2. Memory, including learning and recall [Hopkins Verbal Learning Test-Revised (HVLT-R) and Brief Visuospatial Memory Test-Revised (BVMT-R)]; 3. Abstraction and executive function [Wisconsin Card Sorting Test 64-card version (WCST-64)]; 4. Attention and working memory [continuous performance test-identical pairs (CPT-IP), Wechsler Memory Scale-III (WMS-III), and Paced Auditory Serial Addition Test (PASAT)]; 5. Fine motor skills (Grooved Pegboard Test); 6. Verbal and language (animal-naming tests). The original scores measured in the nine subtests were converted into T-scores in six cognitive domains according to the norm. The activities of the daily living scale were also applied to further confirm whether there was a functional decline in PLWH. Only PLWH received a neurocognitive assessment. Due to incomplete information, the neuropsychological assessment results of four men and three women were not included.

### 2.3. Magnetic resonance imaging data acquisition

All images were collected using a 3.0 T magnetic resonance scanner (Siemens Trio Tim B17 software, Erlangen, Germany) equipped with a 32-channel head coil. During the rs-fMRI imaging data acquisition, subjects were instructed to remain awake and relaxed with closed eyes. T1-weighted structural images were collected using a magnetization-prepared rapid gradient-echo sequence (MRP-RAGE), with a repetition time (TR) = 1,900 ms, echo time (TE) = 2.52 ms, inversion time (TI) = 900 ms, acquisition matrix = 256 × 246, the field of view (FOV) = 250 × 250 mm, flip angle = 9°, and voxel size = 1 mm × 1 mm × 1 mm. Functional images were acquired using a gradient-echo single-shot echo-planar imaging (EPI) sequence, with TR = 2,000 ms, TE = 30 ms, acquisition matrix = 64 × 64, voxel size = 3.5 × 3.5 × 3.5 mm, and flip angle = 90°. A total of 240-time points (35 slices) were collected within 8 min.

The rs-fMRI data were processed using Data Processing & Analysis for Brain Imaging (DPABI) V5.1 (Yan et al., 2016), which is based on Statistical Parametric Mapping.^1^ The preprocessing procedures were as follows: (1) The first 10 time points were removed to minimize the influence of magnetic field instability during the initial scanning. (2) The scanning times of all slices were aligned to the reference slice, using the time of the middle layer as a reference. (3) Head movements were corrected using the realign procedure, and the T1-weighted image was co-registered with the mean of all realigned images. Subjects with an average head movement of >0.2 mm were excluded. (4) The co-registered T1-weighted image was normalized to the Montreal Neurological Institute (MNI) space using the Diffeomorphic Anatomical Registration Through Exponentiated Lie (DARTEL) procedure. The parameters generated in DARTEL registration and normalization were applied to each functional image. (5) The Friston 24-parameter model was used to regress the head motion effects (Friston et al., 1996; Yan et al., 2013). The mean framewise displacement (FD), which was calculated using Jenkinson’s relative root mean square algorithm (Jenkinson et al., 2002), was included in the group analysis as a covariate to further minimize the effect of head motion. A linear detrend was used to reduce high-frequency noise and low-frequency drift. White matter and cerebrospinal fluid signals were also regressed to reduce respiratory and cardiac effects. (6) Temporal bandpass filtering was performed in the slow-5 (0.01–0.027 Hz) and slow-4 bands (0.027–0.073 Hz).

The values of ALFF, ReHo, and VMHC were calculated using DPABI. A fast Fourier transformation was used to convert the time series to the frequency domain for each voxel, and the square root was calculated at each frequency of the power spectrum. ALFF is the average square root of a given voxel. The ReHo was computed using Kendall’s concordance coefficient, which measures the similarity between the time series of a given voxel and its 26 neighboring voxels. The VMHC was calculated as the Pearson correlation coefficient between each pair of mirrored interhemispheric voxel time series. The values of ALFF, ReHo, and VMHC at each voxel were normalized using z-score transformation to improve the reliability and normality across subjects.

In our study, nine seronegative controls and seven PLWHs were excluded because of excessive head movement (mean FD > 0.2 mm). Subsequently, we created a group mask that included all voxels present in at least 90% of participants to ensure that the coverage of these voxels is the same across participants.

### 2.4. Statistical analysis

The general information of the two groups, including age, sex, and years of education, was compared using the two-sample *t*-test (age and years of education) and Fisher’s exact test (sex composition ratio).

Two-way ANOVA was conducted using statistical parametric mapping 12 (see text footnote 1) with the groups (seronegative controls and PLWH) as between-subject factor and different frequency bands as within-subject factors. Age, sex, and head motion were added as covariates. Cluster-level family-wise error (FWE) correction was set at *P* < 0.05. Clusters that survived multiple corrections were extracted as regions of interest (ROIs) for further *post hoc* analysis. The Bonferroni test was used to compare the differences between the two groups at different frequency bands.

Partial correlation analysis was carried out among ALFF, VMHC, ReHo, and each cognitive domain (age and head movement as covariable). *P*-value < 0.05 (not corrected for multiple comparisons) was considered statistically significant.

## 3. Results

### 3.1. Demographic information and clinical variables

There were no statistically significant differences in the age and sex composition ratio between PLWH and seronegative controls (*P* = 0.077 and 0.254, respectively). For PLWH, the viral load, CD4/CD8 ratio, and CD4 + T cell counts were estimated. Among the 98 PLWHs, 66 subjects were tested for plasma viral load within 2 weeks, with 53 subjects showing plasma viral load below the detection limit, 93 subjects were tested for CD4/CD8 ratio, and 98 subjects were tested for CD4 + T cell count. Demographic and clinical information are shown in Table 1.

**TABLE 1:** Demographics and clinical information.

Variable	Controls (*n* = 44)	PLWH (*n* = 98)	*P*-value
Age (mean ± SD)	33.7 ± 6.0	31.6 ± 6.9	0.077a
Male, *n* (%)	40 (91.3%)	94 (98.2%)	0.254b
Education years (mean ± SD)	/	13.5 ± 3.1	/
CD4 + T (mean ± SD)	/	476.6 ± 222.0	/
CD4/CD8 (mean ± SD)	/	0.6 ± 0.4	/
TND, *n* (%)	/	53 (80.3%)	/

TND, virus not detectable. Statistical tests including (a) *t*-test; (b) Fisher’s exact test.

### 3.2. Neurocognitive performance in PLWH

Of the 98 PLWHs, 91 completed neurocognitive tests and activities of daily living scales, and all of them demonstrated intact daily function. For neurocognitive performance, 37 subjects had at least two cognitive domains with at least one standard deviation below the norm and were classified as ANI, 31 had no cognitive deficits in any of the six domains, and 23 had one cognitive domain with at least one standard deviation below the norm. Neurocognitive performance in PLWH is listed in Table 2.

**TABLE 2:** Applied neurocognitive test battery and neurocognitive performance in PLWH.

Test	Cognitive domain	T scores
Trail-making test part A	Speed of information processing	48.4 ± 7.2
Hopkins verbal learning test-revised Brief visuospatial memory test-revised	Memory (learning and recall)	46.8 ± 6.5
Wisconsin card sorting test 64-card version	Abstraction/executive	58.4 ± 9.1
Continuous performance test-identical pairs Wechsler memory scale-III Paced auditory serial addition test	Attention/working memory	44.8 ± 7.2
Grooved Pegboard Test	Fine motor skills	48.4 ± 7.7
Animal naming test	Verbal and language	48.7 ± 7.0

### 3.3. Main effect of group and group-by-frequency interaction of three indices

The main effect of the group can only be detected in the ALFF, with differences located in the right superior/middle/inferior occipital gyrus and right calcarine (extending to the right lingual gyrus and right precuneus; BA18, BA19) (*F* = 25.2, η^2^ = 0.168). Group-by-frequency interactions were detected in ReHo and VMHC. For ReHo, the interaction was found in the right thalamus and right globus pallidus (extending to the right caudate nucleus) (*F* = 24.8, η^2^ = 0.138); for VMHC, the interaction was observed in the bilateral precuneus and bilateral paracentral gyrus (*F* = 18.6, η^2^ = 0.131).

**FIGURE 1:**
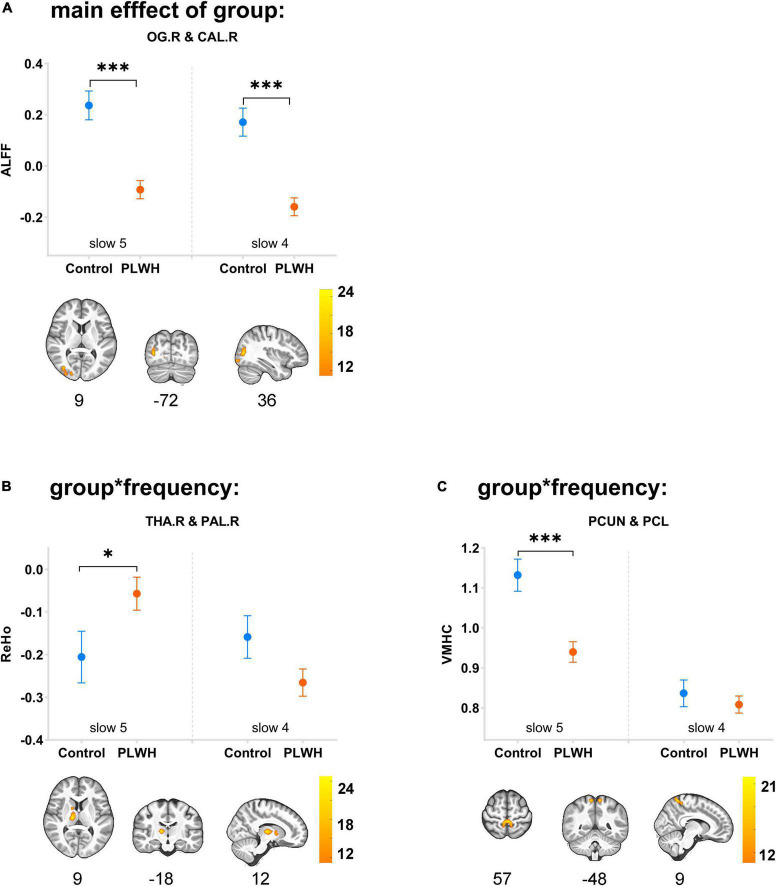
Main effects of group and group-by-frequency interaction in PLWH are compared with seronegative controls. Brain regions showing the main effect of group or group-by-frequency interaction were colored by F-statistic (red/yellow). The upper row graph displayed the differences (mean ± SEM) between groups in slow-5 (plotted on the left side of the separatrix line) and slow-4 (plotted on the right side). ***P < 0.001; *P < 0.05. Corrected for multiple comparisons (*P* < 0.001, corrected at cluster level with FWE *P* < 0.05). OG.R, right superior/middle/inferior occipital gyrus; CAL.R, right calcarine; THA.R, right thalamus; PAL.R, right globus pallidus; PCUN, precuneus; PCL, paracentral lobe; control, seronegative controls; PLWH, people living with HIV.

The subsequent Bonferroni test further supported that the differences in ALFF were independent of frequency bands, with PLWH showing decreased ALFF in the right superior/middle/inferior occipital gyrus and right calcarine (extending to the right lingual gyrus and right precuneus) (*P* < 0.001). The difference in the ReHo of clusters located in the right thalamus/globus pallidus can only be detected in slow-5 with PLWH, showing increased synchronization with nearby voxels (*P* = 0.046). For VMHC, differences could only be detected in slow-5 in the spatially homotopic clusters located in the bilateral precuneus/paracentral gyrus, with PLWH showing decreased synchronization of these mirrored voxels (*P* < 0.001) (see Figure 1 and Table 3).

**TABLE 3:** Main effect of group and group * frequency interaction in PLWH compared with seronegative controls.

Effect	Indices	Structure	L/R	Volumes (mm^3^)	Coordinates			F value
					**X**	**Y**	**Z**	
Main effect (group)	ALFF	Superior/middle/inferior occipital gyrus, calcarine (BA18, BA19)	R	3,915	36	−72	9	25.2
Interaction	ReHo	Thalamus/globus pallidus	R	1,674	12	−18	9	24.8
(group * frequency)	VMHC	Precuneus/paracentral lobe (BA5)	L/R	2,241	9	−48	60	18.6

BA, Brodmann area.

Coordinates (X, Y, and Z) refer to the peak MNI coordinates of brain regions with peak intensity. Corrected for multiple comparisons (*P* < 0.001, corrected at cluster level with FWE *P* < 0.05).

### 3.4. Relationship among ALFF, ReHo, and VMHC indices at different frequency bands and cognition

**FIGURE 2:**
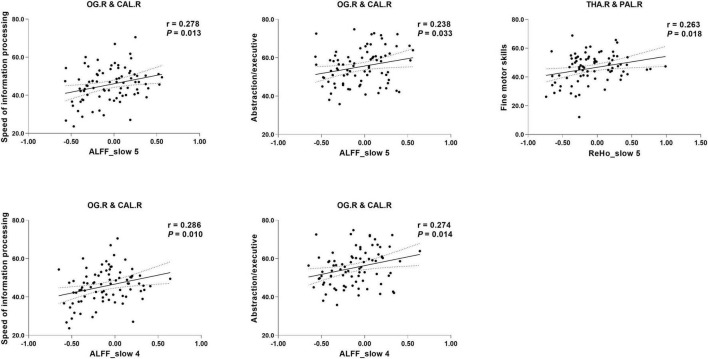
Correlation between specific cognitive domains and imaging indices. The areas between two dotted curves indicate the 95% confidence interval. THA.R, right thalamus; PAL.R, right globus pallidus; PCUN, precuneus; PCL, paracentral lobe.

Partial correlation analysis revealed a significant correlation between the altered indices (except VMHC) and cognition. Specifically, the ALFF of the right occipital gyrus/calcarine in both frequency bands was positively correlated with the speed of information processing (slow-5: *r* = 0.278, *P* = 0.013; slow-4: *r* = 0.286, *P* = 0.010) and abstract/executive function (slow-5: *r* = 0.238, *P* = 0.033; slow-4: *r* = 0.274, *P* = 0.014). The ReHo of the right thalamus/globus pallidus in slow-5 was positively correlated with fine motor skills (*r* = 0.263, *P* = 0.018) (see Figure 2).

## 4. Discussion

In this study, we used three voxel-level fMRI indices (ALFF, ReHo, and VMHC) in two sub-frequency bands (slow-5 and slow-4) to explore functional brain integration and segregation characteristics in neuro-asymptomatic PLWH. Our results indicated that PLWH displayed aberrant functional segregation and integration, including decreased strength of low-frequency oscillations in the right occipital gyrus, increased synchronization of nearby voxels (right thalamus/globus pallidus), and decreased synchronization of mirrored voxels (bilateral precuneus and paracentral gyrus). Applying sub-frequency analysis might be able to detect functional alterations related to the early stages of HAND, and the sub-frequency of slow-5 might be more sensitive in detecting altered intrinsic brain activity than slow-4 in PLWH. Decreased low-frequency fluctuations in the right occipital lobe and increased regional synchronization in the thalamus are associated with impaired cognitive function.

The cortical cortex involved in PLWH detected in our study is mainly the posterior brain cortex, with decreased spontaneous neural activity in the right occipital lobe (mostly in the visual cortex) and reduced neural synchronization of mirrored voxels in the bilateral precuneus/paracentral gyrus. In addition, decreased spontaneous neural activity in the occipital lobe is related to a reduced speed of information processing and abstract/executive skills.

A large body of research supports occipital damage in PLWH (Ances et al., 2009; Wang et al., 2011; Narvid et al., 2018; Wiesman et al., 2018). Wang et al. (2011) indicated abnormal functional connectivity of the external occipital cortex (LOC) network and reduced coactivation of the left inferior parietal cortex in the LOC network 1 year after HIV infection. Ances et al. (2009) found that the primary visual cortex was less activated, and the resting cerebral blood flow in the visual cortex was reduced in PLWH (Narvid et al., 2018). A magnetoencephalography study also revealed aberrant spontaneous and neural oscillatory activity within the visual cortices during a visuospatial processing task (Wiesman et al., 2018). Experimental studies in animals have demonstrated that the occipital cortex is among the most Tat permeable regions (Banks et al., 2005), and this viral protein-induced senescence may contribute to the development of HAND (Marino et al., 2020). In a PET study, higher translocator protein (TSPO), a marker of microglial activation, was detected in the occipital and parietal cortex (Rubin et al., 2018), suggesting pronounced neuroinflammation in this brain region. A similar result was demonstrated by a magnetic resonance spectroscopy study showing cellular inflammation in the occipital cortex and the basal ganglia (Sailasuta et al., 2012). We conjecture that the occipital lobe is likely to be involved in PLWH with prominent inflammation, severe blood–brain barrier disruption, and viral protein accumulation, inducing neurodegeneration, which can be detected by functional imaging. The more prominent the reduction in low-frequency fluctuations, the more pronounced the neuronal damage, resulting in more severe cognitive impairment.

The precuneus is the core area of the default mode network. It shows the highest metabolic rate at rest, has rich anatomical and functional connections with a wide range of brain regions, and participates in multiple complex cognitive functions, such as visuospatial imagination, episodic memory retrieval, and self-task processing (Cavanna and Trimble, 2006). Damage to this brain region was supported by Hall et al. with a task-based functional MRI showing hypoactivation in the precuneus during ambiguous decision-making in PLWH (Hall et al., 2021). In a magnetic resonance spectroscopy study, glutamate reduction in the precuneus of PLWH was reported, and this reduction was associated with information processing speed (Mohamed et al., 2018). In our study, homotopic functional connectivity abnormalities in the bilateral precuneus were detected; however, the reduction was not linked to any of the six assessed domains. One possible reason is the differences in brain reserve and cognitive reserve among PLWHs, which are influenced by neurobiological capacity, genetically determined innate differences, and lifetime exposures (e.g., education, intelligence, and physical activity) (Stern et al., 2019; Zijlmans et al., 2022).

The subcortical nucleus is also involved in PLWH, with increased neural coherence in the right thalamus/globus pallidus. Previous studies have reported thalamic volume loss, even in PLWH with sustained cART (Chiang et al., 2007; Janssen et al., 2015; Wade et al., 2015). Quantitative measurements of [18F] fluorodeoxyglucose (FDG) uptake in PLWH demonstrated that the thalamus showed the most significant hypometabolism (Hammoud et al., 2018). Notably, the subcortical nuclei involved in our study are important components of the cortical-basal ganglia-thalamocortical circuit (Taylor and Taylor, 2000; Chiang et al., 2007). Abnormal quality and quantity of neurotransmitters (decreased dopamine concentration, accumulation of glutamate concentration, and dysfunction of the gamma-aminobutyric acid system) in this circuit were verified by autopsy, cerebrospinal fluid, and animal experiments (Kumar et al., 2009, 2011; Gorska and Eugenin, 2020). In our study, neural synchronization of the right thalamus/globus pallidus was increased in PLWH and positively correlated with fine motor skills. We speculated that the increased coherence in these nuclei might be evidence of brain reorganization in PLWH to maintain cognitive function.

Blood-oxygen-level-dependent (BOLD) signals in slow-4 and slow-5 are considered distinct entities, although the origin and physiological significance of the different sub-frequencies remain unclear. It has been speculated that different frequency bands correlate with different physiological processes (Yang et al., 2020; Liu et al., 2022) and show different properties at the regional, interregional, and network levels (Xue et al., 2014). In our study, group differences were mainly detected in slow-5, indicating that slow-5 might be more sensitive in detecting functional alterations in PLWH, similar to the findings in Alzheimer’s disease (Yang et al., 2020). If only one frequency is analyzed, slow-5 might be a better choice. Previous studies supported that ReHo in slow-4 is enhanced in the ventrolateral thalamus and caudate compared with slow-5 (Xue et al., 2014), and low-frequency fluctuations in slow-4 are more robust in the basal ganglia and the thalamus (Zuo et al., 2010a). However, these studies were conducted in a healthy population; thus, the results may not be generalizable to PLWH. In conclusion, we speculate that the neural activity patterns of some impaired brain regions in PLWH are frequency-specific, and adopting a sub-frequency analysis is helpful in identifying altered functional integration and segregation, which may be related to neurocognition at the early stage of HAND.

Our study had some limitations. First, the neurocognitive function was not observed in seronegative controls. It was reported that 15–22% of seronegative controls score below the threshold of HAND (Cysique et al., 2011). Neurocognitive function in seronegative controls would allow a more objective analysis and conclusion. Second, some risk or causative factors (such as cardiovascular disease, smoking, alcohol abuse, antiviral drug neurotoxicity, and co-infection) were not considered. Third, this was a cross-sectional study, and brain function data before infection or follow-up data were unavailable. Future studies should consider using sub-frequency bands and longitudinal studies to assess cognitive function and brain functional alterations.

## 5. Conclusion

People living with HIV displayed aberrant functional integration and segregation at the early stage of HAND, with altered low-frequency fluctuations in the occipital lobe, enhanced neural synchronization in the right thalamus, and reduced synchronization of mirrored voxels in the precuneus. Most of these alterations are frequency-dependent and are linked to cognitive function. Our study indicates that sub-frequency analysis may show some features that are related to the early stages of HAND and slow-5 might be more sensitive to detect the insidious damage in PLWH. More rigorous study is needed to confirm our findings.

## Data availability statement

The original contributions presented in this study are included in this article/supplementary material, further inquiries can be directed to the corresponding authors.

## Ethics statement

This study was conducted in accordance with the Declaration of Helsinki, and the protocol was approved by the Ethics Committee of Beijing Youan Hospital. The patients/participants provided their written informed consent to participate in this study.

## Author contributions

HL, WW, and DL contributed to the conception and design of the study. WW, YW, RL, JL, and ML recruited the participants and organized the database. DL and WW performed parts of the statistical analysis. WW, DL, and HW contributed to the writing the manuscript. All authors contributed to the manuscript revision and read and approved the submitted version.

## Footnotes

1
http://www.fil.ion.ucl.ac.uk/spm
